# Self-evaluation of automated vehicles based on physics, state-of-the-art motion prediction and user experience

**DOI:** 10.1038/s41598-023-39811-1

**Published:** 2023-08-04

**Authors:** Anne Stockem Novo, Christian Hürten, Robin Baumann, Philipp Sieberg

**Affiliations:** 1 Institute of Computer Science, Ruhr West University of Applied Sciences, Duisburger Str. 100, 45479 Mülheim, Germany; 2 Chair of Mechatronics, University of Duisburg-Essen, Lotharstr. 1, 47057 Duisburg, Germany; 3 Schotte Automotive GmbH & Co. KG, Zum Kraftwerk 1, 45527 Hattingen, Germany

**Keywords:** Computer science, Applied physics

## Abstract

Legal restrictions allow to give full control to automated vehicles for longer time periods either in restricted areas or when moving with reduced speed. Although being technically feasible for a wide range of driving scenarios, the restrictions are still in place due to the lack of a clear safety strategy. An essential step towards safety is the introduction of a self-monitoring component. In this study, a self-evaluation concept is presented which assesses a system based on a physics-defined minimum prediction horizon for state-of-the-art Deep Learning-based trajectory prediction models. Since User Experience is a key metric for car manufacturers, a further manoeuvre constraint is added to the model. We emphasize the generalizability of the presented assessment concept, however, in order to demonstrate feasibility in practical use, three specific scenarios are discussed. The results are gained with real data from publicly available driving campaigns as well as synthetically generated simulation data. Two exemplary models, a simple LSTM-based model and VectorNet, a prominent motion prediction model, are evaluated. A quantitative assessment shows a lack of training data in the public datasets for vehicle speeds > 25 m/s in order to offer safe driving above such vehicle speeds.

## Introduction

Advanced Driver Assistance Systems (ADAS) or SAE^1^ level 1 to level 2 vehicles are already in a mature state. The underlying functions are limited to specific use cases only which is why the development of a validation and verification strategy for such systems is straight-forward. The first SAE level 3 and level 4 vehicles are slowly penetrating the market, however, being constrained to specific environments or situations, e.g. in traffic jams with velocities below 60 km/h, or the permission to operate in confined areas only. SAE Level 3 or *conditional automated driving* still requires a human driver who can take back control upon request. Studies showed that the time until take-over by the human driver is highly dependent on the driver’s state and can easily last more than 10 s^2,3^. This time span has to be safely handled by the system, which is a huge challenge in dense or urban traffic.

Situations that might require a takeover by the driver can be caused by a change in weather or road conditions, e. g. construction sites or the detection of uncommon objects on the road, as well as sensor failures. The Automated Vehicle (AV) might be able to safely circumvent the obstacle, or continue operation in a fail-operational mode until take over by the driver. If this is not possible, a brake to stand still has to be pursued. In order to guarantee that an emergency stop is always possible, the braking time puts a lower constraint on the minimum required prediction time of the traffic situation^4^.

The future traffic situation can be predicted well with Deep Learning models. Such data-driven models show the best performance in anticipating the future situation for several seconds, clearly outperforming physical models^5^. Still, a prediction time of 10 s is challenging and only possible in very few situations, such as straight forward highway driving with moderate vehicle speeds. Especially in urban environments, the prediction error can be huge due to the dynamic scene and often unpredictable behavior of humans. We argue that even for SAE level 3 systems it is not necessary to forecast the entire handover time of 10 s or longer. A high frequent re-planning of the ego trajectory can be done successively and without loss of driving comfort if certain requirements are met. Nevertheless, a frequent update of the current ego manoeuvre might lead to a negative driving experience. For a convenient user experience, comfort aspects can be included in the estimate of the prediction horizon. For example, it shall be guaranteed that a lane change can be executed without having to go back to the initial lane. Therefore, the prediction horizon of a scene forecasting shall exceed the lane change time.

Considering physical laws, the forecasting horizon of the Deep Learning model as well as manoeuvre constraints, we present a general concept for the online safety assessment of an AV system. The purpose of the evaluation system is to know during operation whether the system is safe and comfortable or unsafe, and to be able to inform the driver to be more attentive if required. In case the system is assessed as operating close to the safety limits, the situation shall not occur too long. The driver can then be asked to reduce speed. If the system is not safe, the driver shall be informed immediately that a speed reduction or maintenance is needed if e.g. sensors are not working reliably. The assessment system is presented as a generic concept. In order to demonstrate feasibility of practical use, four specific use cases are discussed, which have been derived with real-driving and simulation data. The introduced time constraints are calculated for state-of-the-art trajectory prediction models. The key takeaway about the proposed solution is that the presented assessment concept is generically applicable and feasible. A quantitative assessment of the system shows a lack of training data in the public datasets for vehicle speeds > 25 m/s in order to offer safe driving above such vehicle speeds.

This paper is structured as follows: In “Related work”, related work is presented followed by a description of the evaluation system and its components in “Model”. The experimental results are presented in “Results”, and the practical use of the assessment system is demonstrated in three example situations on the highway and in urban areas. Finally, in “Discussion” the results are discussed and conclusions are given.

## Related work

According to SAE J3016^1^, AVs’ operations are constrained by their respective Operational Design Domain (ODD). Accounting for environmental conditions like the type of road or the weather as well as vehicle related conditions like the current speed, the ODD defines the operating range in which an automated system may perform the driving task. Reaching or crossing the boundaries of the ODD, the system has to bring the vehicle into a safe state. To prevent the automated systems to fail in case of leaving the ODD, its state has to be monitored and as well be predicted. For example, a system was proposed that supervises the ODD state by forecasting its variables using Bayesian forecasting^6^.

Related work considering the required prediction horizon for SAE level 3 automated vehicles often refers to the take-over time, or *take-over time budget*, required for the driver to regain control over the vehicle and bring it to a safe state^1^. The time budget is often computed by a *time-to-X* metric, most commonly the time-to-collision (TTC) and the time-to-lane-change (TLC)^7^. Having reviewed various studies, the authors conclude that the take-over time budgets are about 8 s on average. It was found that a reduced time budget resulted in a faster, but lower quality, control of the vehicle after take-over. The actual take-over time is dependent on the environment conditions, e. g. the weather or traffic density, as well as the driver state^8^. When finishing non-driving related secondary tasks, drivers may remain distracted up to 27 s reducing their take-over performance^8^. To mitigate the driver’s distraction, an online feedback control was proposed which takes into consideration the driver’s state and warns the driver ahead of a possible take-over request, which finally resulted in a better take-over performance^9^.

An assessment of the prediction horizon via the take-over time was proposed in another study by estimating the execution time for an automated safety action^4^. Emergency braking was considered to be the lower bound for the prediction horizon such that the respective safety action can always be performed. The authors base their concept on the fault tolerant time interval (FTTI) defined in ISO 26262 from 2011^10^ which describes the minimum time span between fault occurrence and the actual hazard. Following the newer version of ISO 26262 published in 2018^11^, the following redefined terms are better suited to represent the underlying concept: emergency operation tolerant time interval (EOTTI), describing the “time-span during which emergency operation can be maintained without an unreasonable level of risk” [ISO 26262 -1 def. 3.44], and emergency operation time interval (EOTI), describing the “time-span during which emergency operation is maintained” [ISO 26262-1 def. 3.45]. In case of emergency braking, the actual braking time makes the major contribution, which is on the order of seconds, while the overhead times, e.g. due to asynchronous update cycles of the system, are on the order of ms^4^. Since the model forecasting horizon is on the order of seconds, the overhead will be neglected in our approach for the sake of simplicity.

State-of-the-art prediction models can forecast the traffic and vehicle state for the duration of the take-over time budgets but are often associated with a high level of uncertainty. It has to be distinguished between data-driven and model-driven approaches. The former use Machine Learning and Deep Learning techniques achieving prediction horizons of up to 8 s while the latter, often physics-based models, can reliably predict less than 2 s^5,12^. A commonly used approach of data-driven models in the field of vehicle motion planning is Deep Reinforcement Learning (DRL). Here, the agent is trained inside a simulation framework to perform (parts of) the automated driving task, including route and trajectory planning, vehicle control and the prediction of changes in the environment. The agent is supposed to find the best driving strategy within the observed environment, often being tuned to multiple objectives (Multi-Objective Deep Reinforcement Learning)^13^. As interactions with the traffic participants are inevitable, the deployment of multiple agents (Multi-Agent Deep Reinforcement Learning) inside one environment is performed to also consider the collaborative nature of driving in traffic^14^. This technique especially proves well in the context of path prediction for Vulnerable Road Users^15^. An alternative approach is modeling the trajectories of traffic participants with supervised learning, using artificial neural networks. Such models take as input several seconds of the past trajectory and output the future trajectory with a forecasting horizon $\Delta t$. The performance of such data-driven forecasting models heavily depends on the specific datasets and conditions which were used during training. Therefore, it is difficult to compare two models against each other. Even comparing the best models from the leaderboard of prediction challenge competitions^16,17^ has to be taken with care since there are several model metrics^18^. These metrics typically vary drastically even for the top models. State-of-the-art forecasting models, such as from the nuscenes competition^16^ aim for prediction horizons of 6 s. The leading model in terms of minimum final displacement error (minFDE = 3.624 m) DGCN_ST_LANE has a miss rate of 46.99 %^16^. The miss rate takes the fraction of predicted trajectories with an offset from the true trajectory at the final trajectory point greater than 2 m. Similarly, for the Argoverse 2 challenge with a prediction horizon of 6 s, the leading model QCNet with minFDE = 1.19 m has a miss rate of 14 %, indicating significant differences in the training data^17^.

These known limitations will be referred to in the results section, “Results”, when calculating specific time constraints for the demonstration of practical use of the entire assessment system.

## Model

**Figure 1:**
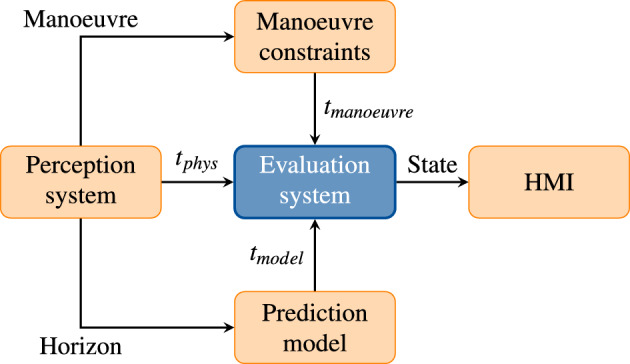
Evaluation system for safe operation of a SAE level 3 AV.

In this section, the generic concept of the AV assessment system is described in detail. An overview of the conceptual design is shown in Fig. 1.

Three contributions are considered as input to the assessment system, a contribution from *physical constraints*, *manoeuvre constraints* as well as the *forecasting horizon* of the prediction model. A comprehensive summary of the input is given in Table 1.

**Table 1:** Description of the input to the evaluation system.

Source	Quantity	Description
Perception system	$t_{phys}$	Constraints from Physics, e.g. emergency braking time, sensor states, weather conditions
Manoeuvre classification	$t_{manoeuvre}$	Constraints from current manoeuvre, e.g. lane change, turn right/left
Trajectory prediction	$t_{model}$	Forecasting horizon of the trajectory prediction model

When assessing the AV operation state, it shall be guaranteed that the system is safe at any time. The additional manoeuvre constraint is introduced since a positive User Experience (UX) is a key performance indicator for car manufacturers. The system shall anticipate with high reliability if a manoeuvre can be terminated successfully after starting the manoeuvre. These constraints are compared against the maximum reliable forecasting time of the prediction model.

The AV is equipped with multiple and often redundant sensors, e.g. Radar, camera, Lidar, Integrated Motion Unit (IMU), Global Positioning System (GPS), for monitoring the system state and the surrounding. During operation, the sensors provide real-time information with update times of a few ms. This information is input to the evaluation system via the physical constraints. The current vehicle speed is needed to calculate the emergency braking time (“Physical constraints”). Further information, such as the weather conditions or location and mapping for determining the environment (urban, rural or highway), can be used in order to refine the physical constraints.

Based on the sensor output, the current driving manoeuvre can be classified as for example straight driving with or without acceleration, turn left/right or lane change. The length of a typical manoeuvre depends on the specific AV and driving situation, which is why it is defined only qualitatively (“Manoeuvre constraints”). For demonstration purposes, a specific use case will be presented in “Results” and quantitative manoeuvre times will be calculated.

The sensor signals are furthermore used as input to a forecasting model for an estimate of the prediction horizon (“Prediction model constraints”). Such a forecasting model shall rely on Deep Learning methods, using either Deep Reinforcement Learning or supervised methods. The specific model details are not of interest at this design stage. Evaluating the prediction model on historic test data, a prediction horizon can be derived and used to compare with the time constraints for braking and manoeuvre duration.

Finally, it is evaluated if the system is operating in the unsafe, safe or comfortable domain. Although being a subjective parameter, the term “comfortable” is limited here to describing comfort as the positive User Experience of guaranteeing the termination of a started manoeuvre. The output of the evaluation system is a variable with the following states:

- 0—*Comfortable operation:* The system operates in a safe and comfortable domain $\rightarrow$ Continue operation
- 1—*Safe operation:* The system operates in a safe domain while manoeuvre constraints are not met $\rightarrow$ Continue operation and monitor state
- 2—*Unsafe operation:* The system operates in an unsafe domain $\rightarrow$ Warn driver to be attentive

The system shall not operate in the safe domain (state 1) for more than $t_{1,thr}$. If the threshold is exceeded, the driver will be asked to take action, e.g. reduce the vehicle speed or clean the sensors. In case the system detects operation in the unsafe domain, the time horizon of the forecasting model is too low, e.g. by driving in an unusual environment, sensor failure, or too high relative speeds. In anticipation of insufficient time in case of takeover, the system warns the driver to be more attentive to the traffic situation.

The derivation of the three time constraints is explained in detail in the following subsections.

### Physical constraints

Scenarios that require an immediate brake to stand still might be the end of a multi-lane road or a roadblock on a single lane. We define a safe situation as such in which the model prediction horizon is greater than the time for an emergency brake. We limit our analysis to the calculation of the braking time via the equation of motion and do not take into account system overhead, stemming from e.g. asynchronous cycles since they are in the range of ms^4^ and can be neglected for our considerations.

#### Dry roads

While the achievable deceleration during emergency braking on a dry road is specific to the vehicle under consideration, in this paper it shall be assumed with the common value of $a_{min}=-8$ m/s$^2$^19^. Assuming that the vehicle is initially cruising with a constant velocity $v_0$ and suddenly has to brake to stand still with approximately constant $a_{min}$, we can derive the velocity as a function of time and set the final velocity to zero:

1 $$\begin{aligned} v(t_{phys}) = v_0 + a_{min} \, t_{phys} = 0 \end{aligned}$$

This sets a minimum time constraint for the prediction model

2 $$\begin{aligned} t_{model} > t_{phys}= \left| \frac{v_0}{a_{min}} \right| \end{aligned}$$

which has to be anticipated for a safe planning.

#### Further road conditions

Different road conditions, e.g. wet or icy road surfaces, can have a strong impact on the effective deceleration. Analogous to Mehmed et al.^4^, we introduce the adhesion coefficient *k* such that maximum achievable deceleration is $a_{min}' = a_{min} k$ with values given in Table 2.

**Table 2:** Maximum achievable deceleration for different road conditions, adapted from^4^.

Road condition	Ice	Snow	Wet slippery	Wet clean	Dry asphalt
$a_{min}'$ (m/s$^2$)	$-1.1$	$-2.3$	$-2.9$	$-5.7$	$-8.0$

The physical time constraint is then

3 $$\begin{aligned} t_{phys}= \left| \frac{v_0}{a_{min}'} \right| . \end{aligned}$$

### Manoeuvre constraints

From the point of safety, it is sufficient to forecast $t_{phys}$. However, if the vehicle has started a manoeuvre, e.g. changing the lane or exiting the highway, it shall be guaranteed that the manoeuvre can be terminated and does not need to be cancelled due to a limited forecasting of the traffic scene. Thus, we include constraints to our model that guarantee the termination of a manoeuvre. The time horizon of the prediction model has to cover the entire duration of a manoeuvre. An additional time constraint is introduced as

4 $$\begin{aligned} t_{manoeuvre} (v_0| {\textbf{X}}_a ), \end{aligned}$$

which is the duration of a manoeuvre depending on a subset $\textbf{X}_a$ of the sensor signals. Please note that the manoeuvre constraint is a soft constraint.

### Prediction model constraints

Given a Deep Learning-based forecasting model, the future position of each traffic participant can be predicted from a subset $\textbf{X}_b$ of the sensor signals,

5 $$\begin{aligned} {\textbf{x}}_i \left( \Delta t \right) = f({\textbf{X}}_b), \end{aligned}$$

with $\Delta t$ the prediction horizon for trajectory *i*. The displacement error $\text {DE}$ is defined as the offset between predicted trajectory ${\textbf{x}}_i$ and its ground truth $\hat{{\textbf{x}}}_i$ as

6 $$\begin{aligned} \text {DE}_{i, \Delta t} = \left| {\textbf{x}}_i \left( \Delta t \right) - \hat{{\textbf{x}}}_i \left( \Delta t \right) \right| . \end{aligned}$$

The reliable prediction horizon per trajectory *i*, $t_{model,i}$ is determined by the time at which the predicted trajectory’s displacement error (DE) first fails to meet the condition DE < 2 m. This criterion is commonly used for the evaluation of state-of-the-art motion prediction models as introduced in the related work section, “Related work”.

The model prediction horizon is finally derived as an ensemble average sampled by the ego speed $v_0$:

7 $$\begin{aligned} t_{model}(v_0) = \frac{1}{N} \, \sum _{i=1}^{N} t_{model, i} \end{aligned}$$

*N* is the number of trajectory samples per velocity range $v_0$.

### Evaluation system

The evaluation system shall constantly assess the three time constraints and send the output state to the Human Machine Interface (HMI) with the following implications.

- *Comfortable operation (State 0):* The prediction model can reliably forecast the time until braking to stand still and the time until the end of the manoeuvre,
  8 $$\begin{aligned} \left( t_{model} \ge t_{phys} \right) \wedge \left( t_{model} \ge t_{manoeuvre} \right) . \end{aligned}$$
  The system operates in a safe and comfortable domain. Vehicle operation can be continued without involving the driver.
- *Safe operation (State 1):* The prediction model can reliably forecast the time until braking to stand still, but does not cover the time until the end of the manoeuvre,
  9 $$\begin{aligned} t_{manoeuvre}> t_{model} > t_{phys}. \end{aligned}$$
  The system operates in a safe domain. Vehicle operation can be continued without any action by the driver but the system state will be monitored. A counter $\Delta t_1$ is started which measures the time for which the vehicle operates in the safe operation domain. The soft constraint shall not be violated longer than $t_{1,thr}$. If the threshold is exceeded ($\Delta t_1 > t_{1,thr}$) the driver is informed to take action.
- *Unsafe operation (State 2):* The prediction model cannot reliably forecast the time until braking to stand still,
  10 $$\begin{aligned} t_{model} < t_{phys}. \end{aligned}$$
  The system operates in an unsafe domain, therefore the driver will be warned to be attentive and monitor the traffic situation.

## Results

In this section, the applicability of the assessment concept shall be demonstrated. Therefore, the three time constraints are calculated using our own simulation data and real driving data from the publicly available Argoverse 2 dataset^17^. For a quantitative assessment of $t_{model}$, we choose a Deep Learning-based supervised approach, in particular, two models with different degrees of complexity: a simple LSTM-based model which is a common approach for handling data sequences, and VectorNet^20^, a more sophisticated model that employs graph representations as a fundamental component. The latter was selected based on its performance (good trade-off between low displacement error and large prediction horizons^5^) and availability of open-source implementations, as it is a prominent model frequently cited in the motion prediction community.

The states ${\textbf{y}}$ of all traffic agents in the scene (a state here refers to positions and velocities of vehicles, pedestrians, cyclists etc.) are predicted by the model as a function

11 $$\begin{aligned} {\textbf{y}}=f({\textbf{X}}_b|\mathbf \theta ), \end{aligned}$$

where ${\textbf{X}}_b$ is a feature vector containing the independent variables (e.g. past agents’ trajectories, static objects, environment parameters). *f* is a transition function determined by the model parameters $\mathbf \theta$, e. g. the weights of a neural network. Details on the model architecture and training can be found in the “Methods” section.

**Figure 2:**
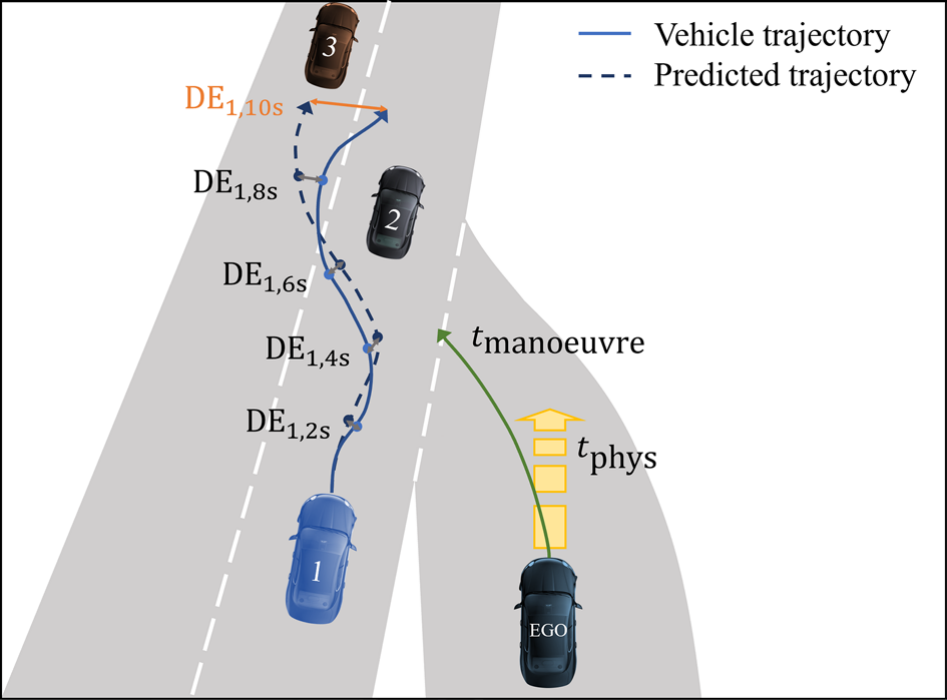
Each predicted vehicle trajectory is compared against its ground truth. The reliable prediction horizon of the model is determined based on the displacement error $\text {DE}_{i, \beta } < 2$ for different prediction horizons $\beta = 2, 4, 6, 8, 10$ s. For vehicle trajectory $i=1$, the maximum prediction horizon is $t_{model, 1} = 8$ s, see Eq. (12).

With the predicted traffic agents’ future positions from Eq. (11) the reliable prediction horizon $t_{model}$ is derived with Eq. (7). The trajectories are sampled discretely with support points $t_\alpha$ and $\alpha = 0,...,A$ as sketched in Fig. 2. Therefore, the reliable prediction horizon per trajectory is obtained as

12 $$\begin{aligned} t_{model,i} = t_{{{\tilde{\alpha }}} - 1} \quad \text { with } \quad \tilde{\alpha }= \underset{\alpha }{\min }\ \left\{ t_\alpha \, | \, DE_{i, t_\alpha } \ge 2 \text { m} \wedge \alpha = 0,...,A \right\} . \end{aligned}$$

**Figure 3:**
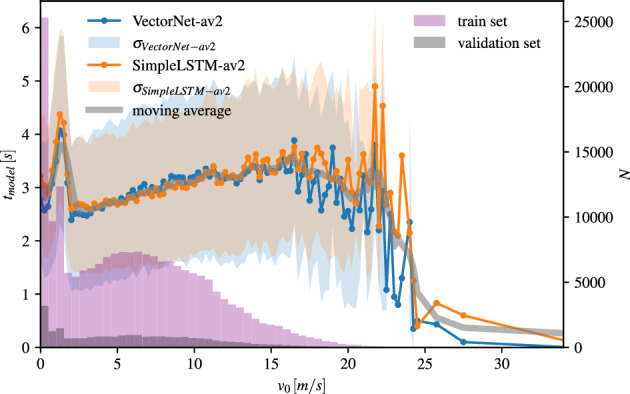
Left y-axis: Trajectory prediction horizon $t_{model}$ with standard deviation $\sigma$ depending on the ego vehicle velocity $v_0$ evaluated on real data (Argoverse 2^17^) for two different prediction models (VectorNet^20^ and simple LSTM). Right y-axis: The number of data samples from the Argoverse 2 dataset per speed range $v_0$ is plotted for the train and validation sets used for training the models.

The real driving data from the Argoverse 2 campaign is shown per velocity bin $v_0$ in Fig. 3 for train and test data. The latter seems to be a good representation of the former, still revealing an oversampling at very low vehicle velocities and an undersampling for vehicle speeds in the range of 1.5–4 m/s. The number of samples at high vehicle speeds (> 25 m/s) decreases rapidly.

The reliable model prediction horizon is shown in Fig. 3 for the simple LSTM model as well as for the VectorNet model. As expected, the model performance reflects the data distribution during training. The prediction horizon peaks at very small vehicle speeds ($v_0 = 1.25$ m/s) and reduces drastically for very large vehicle speeds ($v_0 > 24$ m/s). The displacement error of the individual trajectories varies quite a lot which is reflected by the standard deviation of roughly 1 s for both models. The results are consistent for both models.

**Figure 4:**
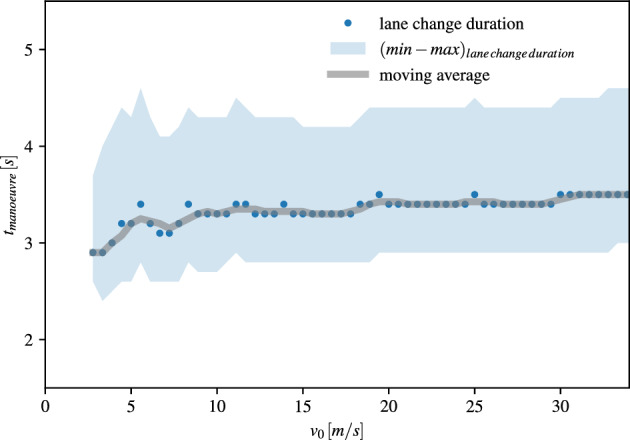
Lane change duration times depending on the ego vehicle velocity evaluated on simulation data with Sumo.

In order to specify the manoeuvre constraints, we demonstrate the application of the assessment system for the use case of lane changes in the urban area with low vehicle speeds and on the highway with intermediate vehicle speeds. The manoeuvre time is derived from simulation of lane changes since this offers more control on the selection of specific scenarios. The manoeuvre time $t_{manoeuvre}$ is defined as the time interval between the beginning of the lateral offset in the origin lane until crossing of the lane marking to the target lane. The details on the simulations are given in the “Methods” section and the results are shown in Fig. 4. Since the simulations do not give plausible results for very low vehicle speeds, the lane change times are given only for vehicle speeds $v_0 > 2.5$ m/s. We observe only a slight increase of the lane change time with increasing vehicle speed. Also for the manoeuvre time, the variance is rather high with more than 1 s above the average value.

Our findings of the lane change times are in agreement with previous studies. In an empirical study the lane change duration in urban environments was found to be approximately 4.2 s^21^ or just 2.5 s for a vehicle speed of 70 km/h^22^. Also on the highway, the lane change time was found to vary between 4 and 8 s^23–26^. The requirement by the ISO 21202 that lane changes on highways have to be terminated within 10 s^27^ is always fulfilled.

**Figure 5:**
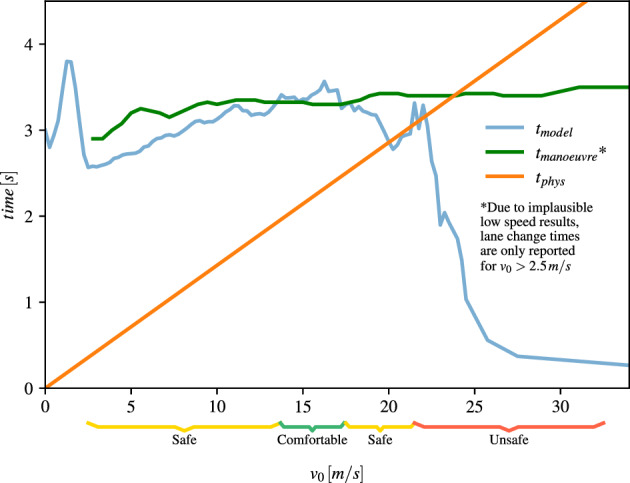
Comparison of the three time constraints $t_{model}$, $t_{phys}$ and $t_{comfort}$ for different vehicle velocities. The situations are tagged by the assessment system as *comfortable*, *safe* or *unsafe* according to Eqs. (8, 9 ,10).

The prediction horizon of the model is compared against the physical and manoeuvre constraints in order to determine the *comfortable*, *safe* and *unsafe* operation domains (see Fig. 5). The standard deviation of the individual curves have been left out for visibility reasons. We observe that there is a wide range of vehicle speed for which the situation is comfortable or at least safe and not much variance is observed in the three time constraints (2.5–20 m/s). For demonstration purposes, we discuss more details for three specific situations.

### Lane change in urban area

Let us assume, an SAE level 3 AV is driving in an urban area with vehicle speed 15 m/s. An impeding vehicle on the ego lane is decelerating and the system triggers a lane change to the left lane. The three time constraints are now compared to each other for this example (see Fig. 5). The time until termination of the lane change manoeuvre was determined in the simulations as $t_{manoeuvre} = 3.2$ s. From the constraint of braking to stand still with $a_{min} = -8 \text { m/s}^2$ the physical time constraint is $t_{phys} = 1.9$ s. From the model performance we derive a reliable time horizon of the prediction model as $t_{model} = 3.2$ s. The driving situation is safe and comfortable because $t_{manoeuvre} \ge t_{model} > t_{phys}$. The current driving situation will therefore be tagged as *comfortable* (State 0).

### Lane change on highway

As a second example, we examine a lane change manoeuvre on the highway. The SAE level 3 AV is driving with a vehicle speed of 25 m/s. An impeding vehicle on the ego lane is the trigger for a lane change to the left lane again. Due to the higher vehicle speed and in agreement with the studies^23–26^, the lane change manoeuvre time is expected to take $t_{manoeuvre} = 3.3$ s. From the constraint of braking to stand still the physical time constraint is derived as $t_{phys} = 3.1$ s. Since Fig. 5 shows a drop in the performance of the forecasting model for high vehicle velocities with a reliable time horizon of $t_{model} = 0.6$ s, the system is operating in the *unsafe* domain (State 2) where $t_{model} < t_{phys}$. In this case, the system shall inform the driver to be attentive and be prepared for a potential take-over of the driving task.

For the training of the models, the dataset Argoverse 2 was used which does not contain a sufficient amount of data for velocities > 25 m/s. Therefore, the model performance at such high velocities is diminished. If such a prediction model would be integrated in the vehicle, the requirements for a SAE level 3 function would not be met for performing lane change maneuvers. In such a situation, the driver should be informed to monitor the driving closely. This information is valuable for the function developer since this means that more data is required for training, especially for velocities > 25 m/s.

The regions where $t_{model}\simeq t_{manoeuvre} > t_{phys}$ are considered as *safe* (State 1) and the counter $\Delta t_1$ is started. If the manoeuvre proceeds as planned, the remaining time until the end of the manoeuvre will soon be low enough, such that the condition $t_{model} > t_{manoeuvre}$ is fulfilled and the evaluation system transitions to State 0. In case, the system remains in State 1 for too long, $\Delta t_1 > t_{1,thr}$, and the driver will be informed by the system to be attentive.

### Interrupted traffic flows on the highway

In very dense traffic on the highway, the flow is interrupted by a frequent stop-and-go at decreased average vehicle speeds. This situation is especially annoying for human drivers since it requires enhanced attention by the driver while performing a monotonous task. Due to the reduced vehicle speed such situations can be handled well by AVs since enough time is given for reaction to changing surroundings. Furthermore, in traffic jams cooperative driving is usually expected. If we assume a vehicle speed of 5 m/s, relation of the three time constraints admits driving in the comfortable domain with $t_{phys} = 0.625$ s, $t_{model} = 3.6$ s and $t_{manoeuvre} = 3$ s.

## Discussion

Conditional automated driving, equivalent to SAE level 3, is especially challenging because the human driver does not need to permanently monitor the traffic and take back the driving task only upon system request. In standard situations, the time span until take-over can last up to 10 s. During this time the system needs to operate at least in a fail-degraded regime. A forecast of the driving situation can therefore help anticipating potential difficulties. The large time horizon until take-over by the human makes such an assessment essential. The more information available about the current driving situation, the more precise will be the determination of the operation domain.

We introduced a generic concept for self-assessment of an AV system and demonstrated feasibility in specific use cases. Three operation domains have been distinguished: (1) comfortable (and safe), (2) safe and (3) unsafe operation. A first finding is that the planning horizon for the ego vehicle path does not need to cover the full time span until take-over, even for operation in the comfortable domain. A frequent re-planning will not be noticed by the passenger if plans are not changed on manoeuvre level. A manoeuvre can be, for example, a lane change or a turn left/right. The minimum time, a forecasting model has to cover is determined by physical requirements. It needs to be guaranteed that the system can always brake to stand still. However, we showed that comfort requirements demand for higher forecasting horizons: For a positive User Experience, it needs to be guaranteed that a started manoeuvre can be terminated successfully and does not need to be cancelled during operation e.g. due to limited perception, sensor failure or difficult lighting conditions. Therefore, the evolution of a traffic scene needs to be anticipated for the time span of the manoeuvre duration. For a comfortable driving experience, the estimated manoeuvre time is a further constraint to be considered. A re-planning of the ego trajectory on time scales of a few ms can then be done successively without the loss of comfort.

Following these observations, three time constraints have been derived: A physical time constraint $t_{phys}$, a manoeuvre constraint $t_{manoeuvre}$ and a time constraint for the time horizon of a motion prediction model $t_{model}$. The physical time constraint has been derived from physics, i.e. the equation of motion. Road surface conditions have been incorporated by an empirical constant that leads to a reduced braking efficiency. Strong emphasis is on the generality of our model, therefore the manoeuvre time constraint and the prediction model constraint have been introduced first as generic concept and have then been discussed for specific implementations. This is beneficial to the community and especially OEMs or Tier 1s who want to assess their implemented driving functions which are often not available to the community. The demonstrated implementations can be easily interchanged.

Simulation data has been used for developing the manoeuvre constraint. For the model constraint, two Deep Learning-based supervised motion prediction models were trained, using the publicly available dataset Argoverse 2. A further finding from our investigations is that this dataset does not contain a sufficient amount of data for velocities > 25 m/s. Therefore, the model performance at such high velocities is diminished. If the motion prediction model solely trained on this data would be integrated in the vehicle, the requirements for a SAE level 3 function would not be met at high driving speeds. In such a situation, the driver should be informed to monitor the driving closely. This information is valuable for the function developer since this means that more data is required for training for velocities > 25 m/s.

The presented lane change use case is discussed for different driving speeds. Also in regions without lane markings, the general concept applies. We are aware of the challenging situation of integrating Vulnerable Road Users (VRUs) since their motion patterns often change on time scales of a fraction of a second. With the presence of VRUs $t_{phys}$ shall still be estimated as the maximum braking time taking of the AV, while taking into account the closest surrounding object, e.g. a VRU. A discussion of lane change time makes sense only for vehicle speeds in the range of 5 m/s or higher. The prediction model performs well at reduced vehicle speeds < 2.5 m/s which is why also in this case the self-assessment would lead to the conclusion of driving in the comfortable domain. However, it should be remarked that our prediction model does not distinguish between the kind of traffic participant. Special attention shall be given to the prediction performance for vulnerable road users in future work. The supervised motion prediction model shall then be exchanged by multi-object Deep Reinforcement Learning, which shows better performance for the integration of VRUs.

A further focus will be on the comfort parameter. Although being a subjective parameter, the term “comfortable” is limited here to describing comfort as the positive User Experience of guaranteeing the termination of a started manoeuvre. The aspects under the term “comfort” can and should be extended in the future. We furthermore plan the implementation of the entire system in simulations and road tests in order to validate the assessment system with real-time estimates.

## Methods

### Training of trajectory prediction model

We investigated two models tasked with predicting the trajectory of an agent to gain insight into the trajectory prediction horizon, as represented by $t_{model}$. The models were trained to output predictions based on a vectorized representation of a given traffic scenario from the Argoverse 2 dataset, which comprises approximately 200,000 training scenarios and 25,000 validation scenarios^17^. Specifically, the goal of these models was to predict the trajectory of the ego agent for the next 6 seconds when given 3 seconds of historical trajectory data of the ego agent as well as all other agents present in the scene in addition to vectorized map information.

The first model examined is an implementation of VectorNet, a neural graph network that processes the spatial relationships of road components and subsequently aims to model the high-level interactions between them^20^. In addition, we also considered a simple encoder-LSTM-decoder network that receives only agent information as inputs. This network consists of three linear layers of 32, 64, and 128 hidden units, each followed by a ReLU activation function for the encoder and decoder, and 3 layers containing 128 hidden units for the LSTM.

Scenes are encoded as a series of polylines $P_j$, with the polylines representing either an agent or a road element. Each polyline is defined as a collection of vectors $v_i\,=\,[ds_i;\,de_i;\,a_i;\,t_i;\,j]$, where *ds* and *de* are the 2D start and end coordinates at time $t_i$, $a_i$ corresponds to the object type of the polyline, and *j* is an integer value representing the polyline ID. The polyline features $a_i$ are discarded for the LSTM model, as this model is intended to make predictions based solely on the coordinates of the agents.

Both models were trained for 25 epochs using the Adam optimizer with a learning rate of 0.001 and a learning rate decay of 0.3 every 5 epochs. Both models were optimized based on the mean squared error.

### Lane change simulation with sumo

Considering the vehicle’s comfort condition, we investigated the required duration for the current driving operation using a simulation, while currently only taking lane changes in account. Therefore, the simulation is carried out as co-simulation between the simulation and numerical computation software MATLAB/Simulink^28^ and the microscopic traffic simulation framework Eclipse SUMO^29^ using the Traffic Control Interface (TraCI) integrated in Matlab by the package TraCI4Matlab^30^.

In our framework, SUMO is responsible for the simulation of the investigated scenario. Due to SUMO’s inaccurate vehicle dynamics modelled by a simple accelerated point-mass approach, the ego vehicle’s dynamics are modelled in Simulink^31,32^. For this purpose, the linearized single-track model is used. This model uses some simplifications to reduce the computational effort, most importantly projecting the wheels of an axle in the vehicles center plane, effectively making it a single-track model, and considering only constant vehicle velocities while disregarding the roll, pitch and lift^33^. The simulation in Simulink is performed with a third order Runge-Kutta-Solver using a fixed step size of 0.01 s.

For the vehicle steering, the used driver model is based on a lateral vehicle guidance approach utilizing the driver’s visual focus point^34^. The driver’s steering therefore is dependent on a visual focus point the driver is focusing on in front of the vehicle. According to the model, the driver will align the vehicle’s driving direction with the line of sight to the specific visual focus point. To execute a lane change, this visual focus point is shifted to the new lane abruptly^35^. For the alignment of the vehicles driving direction respective to the visual focus point, a PID-controller is implemented.

The evaluated lane change time in simulation is the measured time the vehicle needs to move from its starting position in the middle of the lane to the adjacent lane, taking the time when the vehicle completely enters the new lane. The simulations account for varying driver behaviour by modifying the model’s standard steering speed as well as the final lateral position on the lane, which by default would be the middle of the lane.

## Data Availability

The datasets used and/or analysed during the current study are available from the corresponding author on reasonable request.
